# Differential Characteristics and Comparison Between Long-COVID Syndrome and Myalgic Encephalomyelitis/Chronic Fatigue Syndrome (ME/CFS)

**DOI:** 10.3390/biomedicines13112797

**Published:** 2025-11-17

**Authors:** Mariya Ivanovska, Maysam Salim Homadi, Gergana Angelova, Hristo Taskov, Marianna Murdjeva

**Affiliations:** 1 Department of Medical Microbiology and Immunology “Prof. Dr. Elissay Yanev”, Faculty of Medicine, Medical University of Plovdiv, 4002 Plovdiv, Bulgaria; 2 Research Institute at Medical University Plovdiv (RIMU), 4002 Plovdiv, Bulgaria; 3 Laboratory of Clinical immunology, University Hospital St. George, 4002 Plovdiv, Bulgaria; 4 Faculty of Medicine, Medical University of Plovdiv, 4002 Plovdiv, Bulgaria; 5 Department of Microbiology and Virology, Faculty of Pharmacy, Medical University of Pleven, 5800 Pleven, Bulgaria; 6 Institute for Innovation and Smart Technology (IIST), University of Telecommunication and Posts, 1700 Sofia, Bulgaria

**Keywords:** ME/CFS, Myalgic encephalomyelitis, chronic fatigue syndrome, long-COVID

## Abstract

Long-COVID and Myalgic Encephalomyelitis/Chronic Fatigue Syndrome are disabling diseases characterised by ongoing fatigue, post-exertional malaise, cognitive impairment, and autonomic dysfunction. Myalgic Encephalomyelitis/Chronic Fatigue Syndrome typically follows viral infections, whereas Long-COVID exclusively follows SARS-CoV-2 infection, with overlapping but distinct features. This review uses comprehensive searches of online databases to compare their clinical presentations, pathophysiologies, and treatments. Both Long-COVID and ME/CFS appear to involve multifactorial mechanisms, including viral persistence, immune dysregulation, endothelial dysfunction, and autoimmunity, though their relative contributions remain uncertain. Symptom management strategies are consistent, however. Cognitive behaviour therapy has been successful, and there are minimal drug treatments. Graded exercise therapy occupies a contested place, recommending individualised pacing and multidisciplinary rehabilitation. Common and exclusive mechanisms must be identified to formulate valuable therapies. A more significant body of research focusing on immune dysfunction as a pathogenic mechanism for advancing the disease and enabling more effective therapies and diagnostics is needed.

## 1. Introduction

Myalgic Encephalomyelitis/Chronic Fatigue Syndrome (ME/CFS) has been a subject of scientific interest for a very long time, as it is a complex illness. Despite the persistent presence of ME/CFS, the literature has consistently presented it as a disorder that remains shrouded in ambiguity. Numerous studies and scientific articles have explored this illness, often from specialised perspectives, indicating a scarcity of unified information. This suggests that a comprehensive understanding of ME/CFS has yet to be achieved.

Some patients who have gone through the acute phase of infection with COVID-19 have not fully recovered. They develop an array of symptoms, which are collectively described as “Long-/Post-COVID syndrome”. It is defined by the World Health Organisation (WHO) as “the continuation or development of new symptoms 3 months after the initial SARS-CoV-2 infection, with these symptoms lasting for at least 2 months with no other explanation” [1]. There is a resemblance between these symptoms and those typical of the constellation of ME/CFS symptoms.

In the aftermath of the COVID-19 pandemic, the landscape has shifted, offering more specific insights and directing attention towards new avenues of investigation into immunological pathogenesis. Long-COVID has generated renewed interest and attention towards this illness due to the observed parallels in symptoms. This overlap has prompted experts to explore ME/CFS more deeply. ME/CFS has gained prominence because the similarity of its symptoms with those of Long-COVID suggests a new category of disease: infection-triggered chronic illness, described in the literature of PAPIS, PAIS, IACC, and IACI.

Not all patients with Long-COVID exhibit symptoms linked to ME/CFS abnormalities or neurological involvement. This distinction helps in understanding the heterogeneity of Long-COVID, which encompasses a broad range of symptoms. However, many manifestations do not directly involve the central nervous system (CNS), and only a subset of patients exhibit neurological symptoms.

At the same time, post-exertional malaise (PEM) and orthostatic intolerance are the hallmarks of ME/CFS, representing a distinct subset of symptoms. To address their unique symptomatology, patients with Long-COVID who do not fit the ME/CFS criteria might require alternative diagnostic approaches.

We conducted comprehensive research to compare the two conditions—ME/CFS and Long-COVID, to highlight their similar pathophysiology and to propose a research strategy that considers Long-COVID’s viral pathogenesis as a possible etiological mechanism underlying ME/CFS and its associated neuroimmunological features. We searched multiple online databases, including Scopus, Google Scholar, and PubMed/MEDLINE. The key search phrases used were combinations of terms such as “Myalgic Encephalomyelitis” (ME). “Systemic Exertion Intolerance Disease” (SEID), “Chronic Fatigue Syndrome” (CFS), “Chronic Fatigue Immune Dysfunction Syndrome” (CFIDS), and “Post-Viral Fatigue Syndrome” (PVFS). Filters were applied to limit results to studies published between 2018 and 2025 and written in English.

## 2. Pathogenesis, Underlying Mechanisms and Shared Genes in ME/CFS and Long-COVID

ME/CFS is a chronic systemic disease of a disabling nature. When clinicians encounter it and perform standard diagnostic tests, the results are often non-specific. As a result, patients frequently experience prolonged diagnostic delays, consulting multiple specialists over several years before receiving a confirmed diagnosis. Even after diagnosis, there remains a lack of established treatments, contributing to ongoing challenges in clinical management [2]. The absence of reliable diagnostic tests and effective therapies has also led to skepticism regarding the legitimacy of the condition [3]. Machine learning represents a promising tool for both identifying shared features and differentiating patients based on their quantitative and qualitative symptom expression [4]. Some possible diagnostic indicators include oxygen saturation (SpO_2_) and peak body temperature (PBT) measured during the acute phase of COVID-19. These parameters could reflect the level of inflammation and heightened nitro- and oxidative stress, which, in Long-COVID, may correlate with the onset of chronic fatigue symptoms [5].

The central triad of ME/CFS includes fatigue lasting more than six months, non-restorative sleep, and PEM. Rather than resulting from overexertion, even ordinary levels of activity may trigger the onset of post-exertional malaise, which may persist for several days [6]. In addition, orthostatic intolerance, fatigue, flu-like symptoms, pain, sleep changes, and cognitive decline are frequently observed. The illness often progresses gradually, leading to significant disability in many patients [3].

ME/CFS is commonly in patients who have suffered from a viral infection or other types of infectious illnesses and have experienced long-lasting, even chronic, residual fatigue and extreme exhaustion. The factors that worsen the core symptoms, including post-exertional malaise, non-restorative sleep, cognitive impairment, and orthostatic intolerance, include physical exercise, prolonged upright position, and cognitive and emotional stressors [7].

Other hypotheses for the pathophysiology of ME/CFS include suppression of the hypothalamic–pituitary–adrenal (HPA) axis suppression, leading to reduced baseline cortisol levels that may disrupt the regulation of the immune response and contribute to ongoing inflammation. Chronic inflammation and neuroinflammation are potential culprits, as evidenced by laboratory findings and clinical symptoms in affected ME/CFS patients. Moreover, metabolic and genetic defects may contribute to the aetiology of the disease [8].

The development of both Long-COVID and ME/CFS involves generalised metabolic disruptions that cause widespread physiological disturbances across multiple body systems. Over the years, numerous models of ME/CFS pathophysiology have been proposed, each aiming to shed light on its complex nature.

Long-COVID is thought to result from dysregulation of the immune response following an acute SARS-CoV-2 infection [9]. This may trigger autoimmunity, dysbiosis, persistent systemic inflammation, neuroinflammation, and metabolic disturbances.

A prevailing hypothesis suggests that viral infections may be central to the aetiology and pathogenesis of ME/CFS. However, no single underlying trigger or mechanism has been conclusively shown to explain all cases of the syndrome. Nevertheless, one model has been extensively researched, distinguishing itself as a leading proposed cause. A subset of patients has shown upregulation of Epstein–Barr virus (EBV)-induced genes following EBV infection, which encode proteins involved in immune and neurological functions [3]. Other viruses, including cytomegalovirus (CMV), human herpesvirus-6 (HHV-6), and human herpesvirus-7 (HHV-7), have also been investigated [10]. Reactivation of these latent viruses, along with immune dysregulation, may contribute to the clinical manifestations observed in ME/CFS.

To better understand the pathogenesis and affected systems in both diseases, the role of genes has been investigated. The information gathered could be valuable for gaining a deeper understanding of disease mechanisms and could serve as a basis for the development of potential therapeutic agents [11].

**Figure 1:**
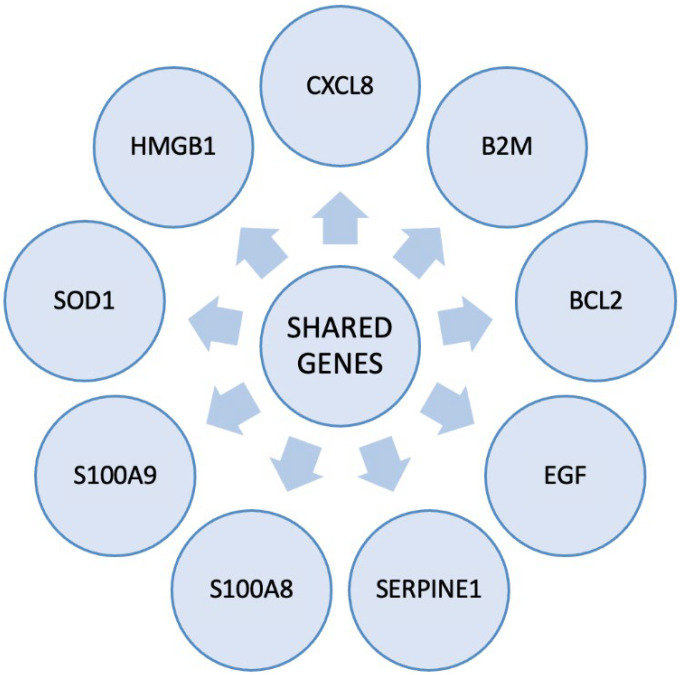
Names of the nine genes that are shared between Long-COVID and ME/CFS. [Made by Maysam Salim Homadi].

Nine shared genes were found between Long-COVID and ME/CFS [11]. These could be associated with leukocyte aggregation on the platelets. These genes include CXCL8, B2M, SOD1, BCL2, EGF, SERPINE1, S100A8, S100A9, and HMGB1, as shown in Figure 1 [11]. The study found that they are tied to peptidyl-cysteine S-nitrosylation, leukocyte aggregation (prevalent in deceased COVID-19 patients) [12], peptidyl-cysteine modification, positive regulation of the intrinsic apoptotic signalling pathway, and response to iron ion [11]. A compelling example worth considering is the role of CXCL8. It has been observed that when CXCL8 expression is higher, IL-8 also increases, which correlates with COVID-19 disease severity [13]. However, it is interesting to note that in 42% of ME/CFS patients, CXCL8 is decreased, whereas IL-8 is increased [14]. Another pertinent example is SERPINE1. SERPINE1 prevents fibrinolysis and contributes to the coagulopathy observed in all COVID-19 patients, regardless of disease severity [15]. Variants in SERPINE1 result in varying levels of coagulation proteins, so fibrinolysis inhibition is associated with thrombophilia. This gene is therefore associated with many diseases where thrombosis is a risk factor [16].

The beta-2-microglobulin (B2M) gene pathway is associated with COVID-19 and other infectious diseases. Elevated B2M levels are correlated with elevated tubular injury markers [17].

SOD1 encodes superoxide dismutase 1, an antioxidant enzyme that destroys free superoxide radicals. Its biological activity provides protection by preventing the overproduction of superoxide radical anions [18].

BCL2 functions as an apoptosis regulator, and its protein has been shown to modulate T-cell activity. When T helper cell immunity is compromised, a depletion process begins, leading to T-cell death through apoptosis. In this context, BCL2 is involved in regulating the intrinsic apoptosis pathway [19].

Epidermal growth factor (EGF) regulates cell growth and differentiation when bound to its receptor. According to Gupta et al. [20], EGF levels are elevated during the moderate stage of COVID-19, and dysregulation of EGF may contribute to disease progression. EGF was also among the 30 soluble factors identified in patients who survived COVID-19, in whom elevated EGF levels were observed, suggesting an association with survival [21].

S100A8 and S100A9 are both calcium-binding proteins whose elevated serum levels are associated with disease severity, as they may modulate cytokine storm responses [22].

Lastly, the shared gene HMGB1 encodes a protein secreted in response to inflammatory signalling pathway and is also associated with COVID-19 disease severity [23].

### 2.1. Immune System Dysfunction in Both Conditions

Regarding immune dysfunction, a side-by-side comparison between Long-COVID and ME/CFS reveals several shared features, as summarised in Table 1. The immune responses in both conditions appear to involve overlapping mechanisms and immunological markers. Recognizing these overlaps and their distinctions may enhance understanding of their pathophysiology and support the development of more targeted treatment approaches.

Lymphocyte changes. In Long-COVID patients, there is marked lymphopenia, a reduced number of dendritic cells, increased activation of CD25+ Th cells, and dysfunction of monocytes. In ME/CFS, a general decrease in cellular immunity is observed, along with increased effector CD8+ T memory cells and decreased terminally differentiated effector CD8+ T-cells [24]. In both diseases, CD8+ cells show either a reduced number (in Long-COVID) or impaired cytotoxicity, and in both, natural killer (NK) T-cells are dysregulated [24]. T-cell exhaustion has also been observed in ME/CFS [7]. Regarding CD4+ cells, both diseases show signs of hyperactivation, with higher Th1, Th2, and Th17 activity in Long-COVID. In contrast, ME/CFS demonstrates a predominant Th2 activation pattern, which may be linked to the autoimmune disturbances observed in patients with ME/CFS [24]. The role of T regulatory cells (Tregs) in the pathogenesis of both conditions should be considered, given their function in providing a balanced immune response. Nonetheless, there has not been a unified answer to their role in the pathology of ME/CFS, with some studies showing elevated Treg levels [25] and others reporting reduced levels [26]. In Long-COVID, Treg dysregulation is also observed, though results remain inconsistent. Some studies report elevated counts, while others show reductions [27]. Given those ambiguous findings, Treg frequency and the Th17/Treg ratio should be further investigated in future studies as potential indicators for better understanding immune regulation in both diseases [27].

Immunologically, it is frequently reported that in ME/CFS, there is an elevation in the number of activated CD8+ T cells, accompanied by a decline in natural killer (NK) cell function [3,28].

In Long-COVID, an imbalance between M1 (pro-inflammatory) and M2 (anti-inflammatory) macrophages has been observed, with a predominance of the M1 subtype [29].

In Long-COVID, B-cell counts are reduced, correlating with disease severity. In ME/CFS, B-cell dysregulation has been reported, which could account for the production of autoantibodies and the subsequent autoimmune responses following chronic viral infection [24].

Cytokine changes. Elevated interferon-α, (IFN-α), tumour necrosis factor-α (TNF- α), granulocyte colony-stimulating factor (G-CSF), interleukin (IL)-17A, IL-6, IL-1β and IL-13 have been measured in Long-COVID patients, consistent with a pro-inflammatory cytokine profile [30]. Higher levels of IL-6 have also been observed in deceased patients [3]. In ME/CFS, pro-inflammatory cytokines such as IL-1, IL-4, IL-5, TNF-α, IL-10, IFN-γ, IL-12, and lymphotoxin-α (LTA) are increased. LTA is a key member of the TNF ligand family, participating in inflammatory responses [31]. IL-6 levels are elevated following physical exertion, which may be linked to PEM in ME/CFS patients [24]. IL-8, IL-16, and tumour necrosis factor-related apoptosis-inducing ligand (TRAIL) are positively correlated to fibromyalgia and gastrointestinal symptoms in ME/CFS [32]. Both syndromes demonstrate an imbalance between pro- and anti-inflammatory cytokine production, which can be viewed as a state of low-grade systemic inflammation or a mild cytokine storm. In multiple sclerosis, elevated IL-6, IL-17, and TNF-α levels have also been positively correlated with chronic fatigue symptoms experienced by patients [33].

Changes in acute phase proteins and hormones. Regarding acute phase reactants, Long-COVID is associated with elevated D-dimer and procalcitonin levels, while ME/CFS is characterised by increased high-sensitivity C-reactive protein (hsCPR) and transforming growth factor (TGF-β) [24]. These markers may be elevated due to viral persistence in Long-COVID and recurrent viral activation in ME/CFS.

ME/CFS patients exhibit dysregulation of the HPA axis [34]. In these patients, the imbalance between Th1 and Th2 responses (favouring Th2) may be influenced by glucocorticoids. Glucocorticoids are steroid hormones that affect various tissues and regulate the circadian rhythm and the stress response. They exhibit pleiotropic effects on the immune system, including suppressing Th1 cell function and promoting the differentiation of Th2 and Th17 cells [35].

**Table 1:** Comparison of the Immunological Changes between ME/CFS and COVID-19.

Markers	ME/CFS	Long-COVID	Implications
CD8+, CD4+	↑ T memory cells, Th2	↑ Th1, Th2, Th17	May reflect ongoing inflammation and autoimmunity
Interleukins	↑ IL-1, IL-4, IL-5, IL-8, IL-10, IL-12	↑ IL-6, IL-8, IL-13, IL-16, IL-17A	Sustained pro-inflammatory cytokine response
TNF	↑ TNF-⍺	↑ TNF-⍺	Promotes inflammation and endothelial activation
Interferons	↑ INF-⍺, INF-r	↑ INF-⍺	Persistent antiviral response, may drive immune exhaustion
TGF–β	↑ TGF–β	–	Suggests immune regulation and fibrosis tendency in ME/CFS
LT⍺	↑ LT⍺	–	Contributes to lymphoid inflammation
G-CSF	–	↑ G-CSF	Reflects chronic inflammation
NK T-cells	↓ NK T-cells	↓ NK T-cells	Impaired viral clearance
APCs	–	↑ B-cells	Maladaptive immune activation
		↓ Dendritic Cells	Indicates impaired antigen presentation
Macrophages	–	↑ M1, ↓ M2	Suggests chronic pro-inflammatory state
Tregs	↑/↓	↑/↓	Potential altered immune regulation

Arrows indicate the direction of change (↑ increase, ↓ decrease). Interpretations are based on key studies referenced in the text [23,24,26,29,30].

### 2.2. Endothelial Dysfunction and Vascular Impairment

Some authors propose a correlation between ME/CFS and abnormal blood flow regulation, suggesting an underlying imbalance in oxygen delivery that leads to tissue hypoxia [36]. This notion contributes to the growing body of evidence supporting endothelial dysfunction in ME/CFS. Endothelial dysfunction is a known cardiovascular risk factor, and many ME/CFS patients have displayed associated cardiovascular symptoms [36]. The study by Scherbakov et al. [37] demonstrated a correlation between peripheral endothelial dysfunction, disease severity, and immune system abnormalities, including the presence of elevated autoantibodies against β2-adrenergic receptors, which are also implicated in the symptoms of ME/CFS [38]. In addition to endothelial dysfunction, hypercoagulability and persistent microclots have been reported in both ME/CFS and Long-COVID. The presence of fibrinaloid microclots in Long-COVID patients may impair oxygen delivery and exacerbate hypoxia-related symptoms. These findings are consistent with altered blood flow and coagulation abnormalities observed in ME/CFS, suggesting a shared pathophysiological mechanism between the two conditions [39].

Both ME/CFS and Long-COVID syndrome have been shown to exhibit endothelial dysfunction [6]. Defective endothelial nitric oxide synthase (eNOS) has been identified in patients with ME/CFS [40], suggesting impaired regulation of vasodilatory mechanisms. The autoimmune processes associated with ME/CFS may further contribute to endothelial impairment [10]. Elevated endothelin-1 levels have been reported in Long–COVID patients, both with and without ME/CFS, and together with defective eNOS, may indicate a vasoconstrictor–vasodilator imbalance [10]. Endothelial damage could contribute to cerebral hypoperfusion [41], which in turn may provoke some of the symptoms observed in ME/CFS, although this mechanism remains to be fully established [10,42].

Moreover, insulin resistance observed in some Long-COVID patients may contribute to endothelial dysfunction, leading to blood–brain barrier disruption and the influx of pro-inflammatory cytokines, which in turn may promote neuroinflammation [43].

### 2.3. Gastrointestinal Tract and Microbiome Disruptions

A large proportion of patients with ME/CFS display gastrointestinal symptoms, particularly inflammatory bowel disease (IBD) [44] and irritable bowel syndrome (IBS), both of which can alter the composition of the gut microbiota [45]. It is, therefore, worthwhile to investigate the gut microbiome for signs of dysbiosis. The microbiome is defined as a “community of microorganisms in a well-defined habitat, structural elements, metabolites, genetic information, and their surrounding environmental conditions” [28]. In ME/CFS, many studies have investigated and reported various alterations, including reduced microbial diversity [46]. Multiple lines of evidence support these findings. For example, elevated levels of potentially harmful bacteria (such as *Streptococcus* spp., *Enterococcus* spp., and *Enterobacterales*) and decreased levels of beneficial bacteria (such as *Bifidobacteria*, the anti-inflammatory *Firmicutes*, and *Bacteroides* spp.) have been described [28,47]. Kitami et al. [45] also reported a reduction in *Faecalibacterium* and an increase in *Coprabacillus*, confirming findings from earlier studies. Zhou et al. [48] observed elevated levels of *Actinomyces* in ME/CFS patients. Expanding beyond the gut microbiome, Lupo et al. [46] emphasise the importance of not disregarding the oral microbiome, as it can stimulate pro-inflammatory cytokines that may affect neurocognition. In general, it should also be considered whether the medications that ME/CFS patients regularly take may significantly influence their microbiota [28].

Long-COVID patients exhibit a greater risk of developing IBS compared with non-infected individuals. A meta-analysis that excluded studies lacking control for pre-exiting gastrointestinal diseases supports this statement. However, it also highlights a significant difference between Long-COVID patients assessed for IBS within 12 months of the acute infection and those evaluated beyond this period, with the latter group showing a markedly lower incidence of IBS [49].

In ME/CFS and Long-COVID, gastrointestinal symptoms may be influenced by the specific pathogen involved. SARS-CoV-2 is known for its adverse effects on the gastrointestinal tract. Three plausible mechanisms for virus-induced gut dysbiosis have been proposed: intestinal inflammation, angiotensin-converting enzyme 2 (ACE2) dysregulation, and bacteriophage-like behaviour. The virus binds to ACE2 receptors on enterocytes, enters the cells, activates the immune response, upregulates pro-inflammatory mediators, and induces inflammation. It may also penetrate the gut microbiota and act as a bacteriophage, disrupting microbial balance [50]. Alterations in the microbiome and colonic inflammation increase gut permeability. This permits the translocation of lipopolysaccharides (LPS) [51] and peptidoglycans (PGN) [52] into the circulation, where they induce systemic inflammation, cross the blood–brain barrier, promote neuroinflammation, and contribute to cognitive symptoms. In ME/CFS, mechanisms such as increased gut permeability and microbial translocation may explain elevated IgA and IgM responses to LPS in peripheral blood, as well as the gastrointestinal symptoms commonly observed [28]. In Long-COVID patients, the persistence of viral nucleic acid within tissues has been documented and may predict the likelihood of developing Long-COVID symptoms. An inverse relationship has been established between the time elapsed since acute infection and the percentage of patients with persistent viral load [53].

In addition to microbiome-related mechanisms, another line of evidence implicates the autonomic nervous system (ANS) in gastrointestinal pathogenesis. This hypothesis has emerged from observations of autonomic imbalance and altered gut motility in patients with ME/CFS and Long-COVID. The sensory branch of the ANS may detect systemic inflammation and, through the CNS, stimulate the HPA axis [54]. Disruption of the HPA axis could have a significant effect on gastrointestinal function. Glucocorticoids are linked to modulation of gut microbiome and alterations in colonic permeability. Therefore, a neuroendocrine dysfunction may disrupt the gut–brain axis, contributing to neurological and psychological symptoms in both diseases [55]. Another plausible pathway involves elevated serotonin levels during high-stress periods, accompanied by surges of dopamine and noradrenaline, which may precipitate gastrointestinal disturbances [54].

### 2.4. Metabolic and Mitochondrial Dysfunction

Dysfunctional mitochondria in Long-COVID patients and ME/CFS patients are associated with impaired oxidative phosphorylation, defective energy metabolism, and reduced ATP-linked respiration [56,57]. Mitochondrial damage in gut and immune cells may play an important role in the dysregulation of circadian rhythms observed in patients with chronic fatigue syndrome [24]. A redox imbalance identified in both syndromes contributes to a defective tricarboxylic acid (TCA) cycle, decreased fatty acids and acyl-carnitines, and compensatory alterations in glycolysis (either elevated or reduced), ultimately leading to energy metabolism disturbances and mitochondrial dysfunction. An imbalance between pro-oxidants and antioxidants is also evident in ME/CFS: pro-oxidants such as peroxides and superoxides are elevated (correlating with symptom severity), nitrosative stress is increased (with activation of NF-κB pathway, also observed in Long-COVID patients) [11], and antioxidants levels—particularly vitamin E—are reduced [7]. The study by Al-Hakeim et al. [57] highlights the importance of an imbalance between oxidative and antioxidative metabolites, demonstrating lower glutathione peroxidase and zinc levels, along with increased myeloperoxidase and nitric oxide production in Long-COVID patients experiencing chronic fatigue symptoms.

A typical trait of patients with ME/CFS is a hypometabolic state, which may result from uneven energy distribution toward the synthesis of pro-inflammatory cytokines and cytokine-storm-like immune reactions. Several patients with acute COVID infection have shown regional cerebral hypometabolism, particularly in certain cortical regions, correlating with cognitive dysfunction and a heightened inflammatory response. Over time, this hypometabolic state appears to diminish and may eventually resolve [58].

ME/CFS and Long-COVID may share a common mechanism of mitochondrial damage, including altered glucose metabolism, impaired oxidative phosphorylation (OXPHOS), and a compensatory shift toward anaerobic glycolysis to maintain cellular energy supply [59,60]. Proposed biomarkers for those metabolic processes include elevated lactate [59,60] and reduced pyruvate dehydrogenase activity [61].

### 2.5. Nervous System Dysfunction

ME/CFS patients frequently report cognitive impairments, with common difficulties including slowed information processing, poor memory function, mental fatigue, and reduced thinking speed [62,63]. Proposed mechanisms underlying these cognitive deficits include “immune abnormalities, presence of antibodies, orthostatism, breakdown of the blood–brain barrier or neuroinflammation” [64]. Similarly, Long-COVID patients experience persistent symptoms such as brain fog and fatigue following the acute phase of infection.

**Figure 2:**
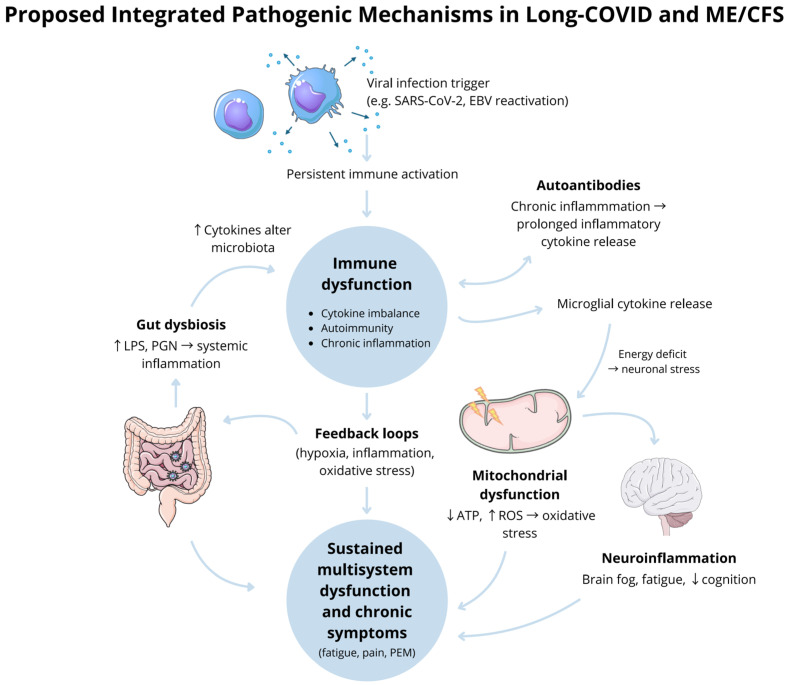
Proposed Pathogenic Mechanisms Underlying Long-COVID and ME/CFS. Following viral infection, persistent immune activation leads to cytokine imbalance, autoimmunity, and chronic inflammation. Key proposed mechanisms driving chronic symptoms in both Long-COVID and ME/CFS include mitochondrial dysfunction, vascular impairment, neuroinflammation, immune dysregulation, and gut dysbiosis, all contributing to disease pathology. These interconnected pathways sustain multisystem dysfunction characteristics of Long-COVID and ME/CFS. Created using images from [74]. [Made by Maysam Salim Homadi and Gergana Angelova].

Azcue et al. [64] analysed and compared the cognitive performance and neuropsychiatric symptoms of ME/CFS and Long-COVID patients. They found that the most prevalent symptoms shared by both conditions were PEM, sleep disturbances, and mental fatigue. Sustained attention capacity was impaired in both groups but was slightly more affected among ME/CFS patients [64]. Mantovani et al. [65] reported that up to 27% of the patients in their study exhibited ME/CFS-like symptoms persisting for more than six months after recovery from COVID-19. Vagus nerve inflammation has been implicated as a potential contributor to autonomic dysfunction in Long-COVID, according to recent findings [66]. Additionally, dysautonomia, including Postural Orthostatic Tachycardia Syndrome (POTS), has been observed in both Long-COVID and ME/CFS patients. This may result from immune dysregulation, endothelial dysfunction, and vagus nerve impairment, leading to cardiovascular symptoms such as orthostatic intolerance and tachycardia [67]. Small fibre neuropathy (SFN) has also been reported in both Long-COVID and ME/CFS patients, potentially contributing to sensory abnormalities and autonomic dysfunction [68]. The interconnected mechanisms proposed to link viral infection with immune dysregulation, mitochondrial dysfunction, endothelial impairment, and neuroinflammation are illustrated in Figure 2. 

ME/CFS is associated with neuroinflammation [69]. Microglial activation has been identified in Long-COVID, with neuropathological findings indicating neuroinflammatory changes particularly affecting the brainstem and cerebellum [70], which may contribute to cognitive dysfunction and fatigue. Neuroinflammation could therefore represent one of the mechanisms underlying the cognitive disturbances observed in these patients. Elevated levels of pro-inflammatory cytokines, including IL-1, IL-6, and TNF-α, have also been reported in patients with mood and anxiety-related disorders [71]. In contrast, some studies suggest that Long-COVID-related brain alterations are not associated with active neuroinflammation, but rather with peripheral inflammatory mechanisms [7,72].

Furthermore, an imbalance in the kynurenine/tryptophan ratio, likely resulting from greater systemic inflammation in severe Long-COVID cases, may be linked to the observed neurotoxic consequences [73].

## 3. Treatment and Management Approaches

More studies are needed to address the marked heterogeneity among patients with ME/CFS and Long-COVID, as well as intragroup variability, despite their seemingly similar pathophysiology. Patients within these groups often differ in presentation and treatment response, suggesting that the understanding of their pathogenesis remains incomplete and poorly unified. Alternatively, these pathological states may arise from diffuse imbalances across multiple systems, with variations in the pathways of dysfunction that ultimately converge into similar symptom profiles. Because of the difficulty in pinpointing a specific mechanism to target as a primary treatment, no conclusive data currently support a standardised therapeutic regimen. In light of these challenges, the following study [75], which evaluates the effectiveness of several treatment approaches, provides valuable insights and general guidance for selecting management strategies for these complex conditions.

Disease severity appears to be the most influential factor affecting treatment effectiveness. However, variables such as sex, age, disease duration, and diagnostic status also seem to significantly influence treatment outcomes, sometimes even more so than the diagnosis of ME/CFS or Long-COVID itself. This observation suggests that individual variability plays a major role in the presumed convergent pathogenesis. In general, Long-COVID patients may be more responsive to the proposed therapeutic interventions.

To better address the diverse therapeutic needs of patients with either condition, it may be useful to classify them according to their predominant symptoms. The study found that the most consistently beneficial interventions across all symptom clusters were activity pacing and fluid/electrolyte management. Other therapies, recommended according to specific symptom clusters, are summarised as follows:

- Cluster 1—Multisystemic Symptomatology: Treatment approaches include manual lymphatic drainage and intravenous or subcutaneous immunoglobulin (IgG) therapy to address immune dysfunction.
- Cluster 2—POTS-Dominant Presentation: Activity pacing and the use of compression stockings are recommended. The use of compression stockings may help manage orthostatic intolerance.
- Cluster 3—Cognitive and Sleep Dysfunction with Increased Pain: Activity pacing and ADHD-type medications may be beneficial for the management of brain fog and neuropsychiatric symptoms.
- Cluster 4—Milder Symptomatology: Activity pacing is recommended to manage PEM and stabilise energy levels.

The study [75] has its limitations; however, it may serve as a valuable foundation for designing future research on ME/CFS and Long-COVID, particularly studies focusing on distinct patient subgroups within each condition.

Moreover, the neuroinflammation, gut dysbiosis, and associated neuropsychiatric symptoms observed in both conditions may be mitigated through probiotic therapy. Restoring gut microbial balance, improving barrier function, and reducing intestinal permeability could represent important steps toward managing these chronic, long-lasting conditions [76]. Nevertheless, further studies are required to establish definitive efficacy, and an individualised treatment approach remains advised.

## 4. Conclusions

The onset of ME/CFS after a prior infection and its possible evolution after a SARS-CoV-2 infection suggest a possible shared pathogenesis with Long-COVID, especially considering the similarities in the clinical symptoms. Therefore, a distinction should be made in those cases based on the patient’s history and possible immunological markers.

Long-COVID syndrome and ME/CFS are conditions with overlapping symptoms and potentially converging pathophysiological mechanisms. Both conditions manifest as complex, multisystem illnesses, often with fatigue, post-exertional malaise, and cognitive dysfunction as hallmark features. While ME/CFS has long been poorly defined, the emergence of Long-COVID syndrome offers an opportunity to study chronic illnesses with a defined initiating event, in this case, SARS-CoV-2 infection.

Key parallels, such as immune dysregulation, neuroinflammation, and metabolic disturbances, provide a framework for shared investigation. However, the distinctiveness of each condition must not be overlooked, mainly as only a subset of Long-COVID patients fulfil diagnostic criteria for ME/CFS. Understanding these overlaps and differences will be instrumental in refining the diagnostic tools and treatment approaches.

Further research is needed to elucidate the shared and divergent pathways, focusing on immune markers, mitochondrial dysfunction, and the gut–brain axis. These insights could pave the way for targeted therapies and advancing care for ME/CFS patients and patients with long-term sequelae of Long-COVID.

## Data Availability

No new data were created or analyzed in this study.

## Abbreviations

ANS	Autonomic nervous system
ATP	Adenosine triphosphate
BCL2	B-cell lymphoma 2
B2M	Beta-2-microglobulin
CD8+	Cluster of differentiation 8 positive
COVID-19	Coronavirus disease-19
CMV	Cytomegalovirus
CNS	Central nervous system
CXCL8	C-X-C Motif Chemokine Ligand 8
EBV	Epstein–Barr Virus
EGF	Epidermal Growth Factor
ENOS	Endothelial nitric oxide synthase
G-CSF	Granulocyte colony-stimulating factor
HHV-6	Human herpesvirus 6
HHV-7	Human herpesvirus 7
HMGB1	High mobility group box 1 protein
hsCPR	High sensitivity C-reactive protein
IBD	Inflammatory bowel disease
IBS	Irritable bowel syndrome
IFN-γ	Interferon-γ
IL-10	Interleukin 10
LTA	Lymphotoxin- α
LPS	Lipopolysaccharides
ME/CFS	Myalgic Encephalomyelitis/Chronic Fatigue Syndrome
NF-κB	Nuclear factor kappa B
NO	Nitric oxide
NK cells	Natural killer cells
OXPHOS	oxidative phosphorylation
PGN	Peptidoglycans
PEM	Post-exertional malaise
SARS-CoV-2	Severe acute respiratory syndrome coronavirus-2
SERPINE1	Serpin Family E Member 1
SOD1	Superoxide dismutase type 1
S100A8	S100 calcium-binding protein A8
S100A9	S100 calcium-binding protein A9
TCA cycle	Tricarboxylic acid cycle
TGFB	Transforming growth factor beta
Th cells	T-helper cells
TNF-α	Tumor necrosis factor-α
TRAIL	Tumor necrosis factor (TNF)-related apoptosis-inducing ligand
Tregs	T regulatory cells
WHO	World Health Organization
